# A Continuous-Wave EPR Investigation into the Photochemical Transformations of the Chromium(I) Carbonyl Complex [Cr(CO)_4_bis(diphenylphosphino)]^+^ and Reactivity with 1-hexene

**DOI:** 10.3390/molecules29020392

**Published:** 2024-01-12

**Authors:** David Fioco, Andrea Folli, James Platts, Mario Chiesa, Damien M. Murphy

**Affiliations:** 1 School of Chemistry, Cardiff University, Main Building, Park Place, Cardiff CF10 3AT, UK; follia@cardiff.ac.uk (A.F.); platts@cardiff.ac.uk (J.P.); 2 Dipartimento di Chimica e NIS Centre, Università degli Studi di Torino, via P. Giuria 7, 10125 Turin, Italy; mario.chiesa@unito.it

**Keywords:** EPR spectroscopy, photochemistry, chromium (I), paramagnetic, phosphines

## Abstract

Chromium complexes containing a bis(diphenylphosphino) ligand have attracted significant interest over many years due to their potential as active catalysts for ethylene oligomerisation when combined with suitable co-catalysts such as triethylaluminium (TEA) or methylaluminoxane (MAO). While there has been considerable attention devoted to the possible reaction intermediates and the nature of the Cr oxidation states involved, the potential UV photoactivity of the Cr(I) complexes has so far been overlooked. Therefore, to explore the photoinduced transformations of bis(diphenylphosphino) stabilized Cr(I) complexes, we used continuous-wave (CW) EPR to study the effects of UV radiation on a cationic [Cr(CO)_4_(dppp)]^+^[Al(OC(CF_3_)_3_)_4_]^−^ complex (**1**), where dppp represents the 1,3 bis-(diphenylphosphino)propane ligand, Ph_2_P(C_3_H_6_)PPh_2_. Our preliminary investigations into the photochemistry of this complex revealed that [Cr(CO)_4_(dppp)]^+^ (**1**) can be readily photo-converted into an intermediate *mer*-[Cr(CO)_3_(*κ*^1^-dppp)(*κ*^2^-dppp)]^+^ complex (**2**) and eventually into a *trans*-[Cr(CO)_2_(dppp)_2_]^+^ complex (**3**) in solution at room temperature under UV-A light. Here, we show that the intermediate species (**2**) involved in this transformation can be identified by EPR at much lower temperature (140 K) and at a specific wavelength (highlighting the wavelength dependency of the reaction). In addition, small amounts of a ‘piano-stool’-type complex, namely [Cr(CO)_2_(dppp-*η*^6^-arene)]^+^ (**4**), can also be formed during the photoconversion of [Cr(CO)_4_(dppp)]^+^ using UV-A light. There was no evidence for the formation of the [Cr(L-bis-*η*^6^-arene)]^+^ complex (**5**) in these UV irradiation experiments. For the first time, we also evidence the formation of a 1-hexene coordinated [Cr(CO)_3_(dppp)(1-hexene)]^+^ complex (**6**) following UV irradiation of [Cr(CO)_4_(dppp)]^+^ in the presence of 1-hexene; this result demonstrates the unprecedented opportunity for exploiting light activation during Cr-driven olefin oligomerisation catalysis, instead of expensive, difficult-to-handle, and hazardous chemical activators.

## 1. Introduction

Ethylene oligomerisation is a commonly employed process for the large-scale production of linear alpha-olefins (LAOs) [1], i.e., important precursors for the production of low-density polyethylene, surfactants, and synthetic lubricants. Organo-chromium complexes with bis(phosphino)amines or bis(sulfanyl)amines ligands have been employed for the specific production of 1-hexene and 1-octene from ethylene feedstocks, as these complexes appear to prevent a very wide product distribution and have shown a particularly high selectivity for 1-hexene [2,3,4,5,6,7,8,9]. The general consensus is that catalysis occurs via a metallacyclic mechanism [10,11], although detailed mechanistic insights into the reaction cycle still remain largely elusive, particularly with respect to the chromium oxidation state(s) that are mostly responsible for the macroscopic catalysis observed following chemical activation with methylaluminoxane (MAO) or triethylaluminium (TEA) [12].

Combined EPR, XAS, and computational studies have recently been employed to elucidate these challenging, oxidation state-related, mechanistic details [13,14,15]. Depending on the nature of the catalysts and the activator used, chromium (I), (II), and (III) oxidation states have all been invoked as potential catalytically relevant oxidation states, leading to controversy and contrasting views in the scientific community. Earlier investigations showed that cationic Cr(I) complexes in the form of [Cr(CO)_4_(Ph_2_PN(R)PPh_2_)]^+^ (where Ph_2_PN(R)PPh_2_ is normally abbreviated to PNP) in the presence of a weakly coordinating anion, such as [Al(OC(CF_3_)_3_)_4_]^−^ along with excess AlEt_3_, can also successfully catalyse ethylene tri- and tetra-merization [4,16]. During catalysis, the carbonyl ligands must be removed to enable ethylene coordination and oxidative coupling from the metallacycle intermediate, highlighting the importance of the co-catalyst as an efficient decarbonylating agent [4,16] of the [Cr(CO)_4_(Ph_2_PN(R)PPh_2_)]^+^ pre-catalyst complex. It is thought that MAO reacts with the Cr precatalyst to effectively produce the cationic catalytically active species [4]. The required high MAO to Cr ratios and high costs of MAO made this path undesirable, so alternative co-catalysts such as TEA were considered. The decarbonylation reaction was indeed found to proceed rapidly in the presence of TEA, resulting in the formation of several different varieties of Cr(I) complexes, including Cr(I)-bis-η^6^-arene [17] and ‘piano-stool’ [Cr(CO)_2_(L)]^+^ [18] complexes, as evidenced and fully characterised by EPR spectroscopy.

Rucklidge et al. [4] also showed that the UV irradiation of a Cr(I) complex in solution could result in the loss of the carbonyl IR bands associated with the cationic [CrCO_4_(PNP^iPr^)]^+^ starting complex, with the concomitant formation of new carbonyl bands suggestive of the formation of a Cr(0) complex, whilst also indicating (from quantitative analysis of the data) that some of the Cr centres no longer possessed CO ligands. Interestingly, UV decarbonylation alone did not produce an active catalyst from the cationic [Cr(CO)_4_(PNP^iPr^)]^+^ complex unless, as proposed, a scavenger species was present to permanently remove the carbonyl ligands from the chromium centres [4].

In light of this evidence, we recently studied the photochemistry of the cationic complex [Cr(CO)_4_(Ph_2_P(C_3_H_6_)PPh_2_)]^+^ (where Ph_2_P(C_3_H_6_)PPh_2_ refers to 1,3 bis-(diphenylphosphino)propane, and is commonly abbreviated to dppp), following UV irradiation in order to identify and characterise the nature of any paramagnetic complexes formed, ascertaining the potential application of UV-A light as an alternative to TEA for the decarbonylation of [Cr(CO)_4_(dppp)]^+^ during ethylene tri- and tetra-merization catalysis [19]. Work by Sasol has shown that this complex does produce an active catalyst for ethylene oligomerization [4,20]. Indeed, the photophysics, photochemistry, and the photoelectrochemistry of structurally equivalent metal carbonyl complexes have been well characterised in the past [21,22,23,24]. For the Cr(I) centres, upon UV irradiation at room temperature, we observed the transformation of [Cr(CO)_4_(dppp)]^+^ into a homoleptic *trans*-[Cr(CO)_2_(dppp)_2_]^+^ complex via EPR spectroscopy. This transformation was also accompanied by a significant conversion of Cr(I) into an unidentified EPR-silent compound(s), as highlighted by the semi-quantitative analysis performed [19]. At lower temperatures (77–120 K), however, EPR spectroscopy of UV-irradiated [Cr(CO)_4_(dppp)]^+^ revealed the presence of a transient *mer*-[Cr(CO)_3_(*κ*^1^-dppp) (*κ*^2^-dppp)]^+^ complex [19], which could be further decarbonylated to generate, once again, the *trans*-[Cr(CO)_2_(dppp)_2_]^+^. This evidence is in line with the work of Rieger et al., who demonstrated the UV light-driven decarbonylation of an electrochemically generated *mer*-[Cr(CO)_3_(*κ*^1^-L_2_)(*κ*^2^-L_2_)]^+^ complex (L_2_ = bidentate phosphine, abbreviated to dppe) using EPR spectroscopy [25]. We showed that these photochemical transformations are concentration-dependent, involving a bimolecular mechanism, which accounts for the formation of *mer*-[Cr(CO)_3_(*κ*^1^-dppp) (*κ*^2^-dppp)]^+^ and *trans*-[Cr(CO)_2_(dppp)_2_]^+^ along with indirect evidence for the additional formation of EPR-silent Cr species and Cr(I) species in solution based on the EPR analysis [19], similar to the findings of Rucklidge et al. [4].

Owing to the importance of the many experimental variables in this photochemistry, including sample concentration, temperature, wavelength, and power, we sought to further explore the effects of UV irradiation on this important class of [Cr(CO)_4_(dppp)]^+^ complexes using EPR spectroscopy and, in particular, to identify any new complexes formed following the addition of 1-hexene to the UV-treated complex.

## 2. Results and Discussion

### 2.1. Overview of Cr(I) Photochemistry for the Cationic [Cr(CO)_4_(dppp)]^+^[Al(OC(CF_3_)_3_)_4_]^−^ Complex *(**1**)*

**Figure 1:**
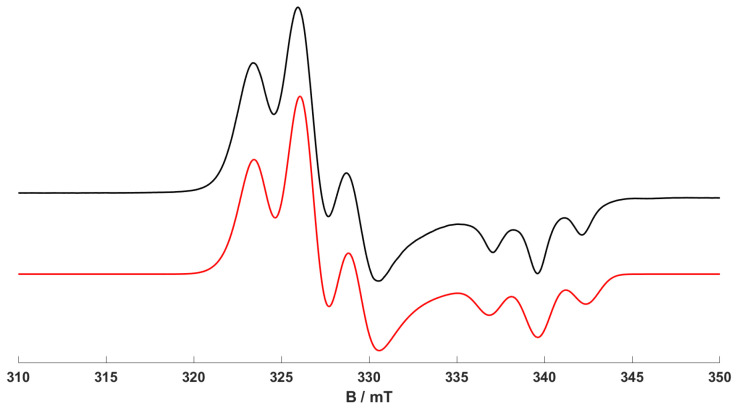
CW X-band EPR spectrum of the [Cr(CO)_4_(dppp)]^+^ complex (**1**) recorded at 140 K (using 1 mM of (**1**) dissolved in dichloromethane, abbreviated to DCM). Experimental shown in black, with corresponding simulation shown in red.

The CW X-band EPR spectrum of the starting [Cr(CO)_4_(dppp)]^+^ complex (**1**) is shown in Figure 1. A similar series of EPR spectra was previously reported by us for structurally analogous Cr(I) bis(diphenylphosphino)-type complexes [16]. Therefore, only a brief overview of the spin Hamiltonian parameters for this complex will be presented here, in order to clearly distinguish these parameters for (**1**) from those of the newly formed complexes presented in this work. The frozen solution CW EPR spectrum of (**1**) is characterised by an axial *g* profile and well-resolved super-hyperfine structure arising from the two equivalent ^31^P nuclei (*I* = 1/2) of the dppp ligand, which are responsible for the 1:2:1 intensity pattern. Satellite resonances arising from the hyperfine coupling of the unpaired electron to the ^53^Cr nucleus (*I* = 3/2, natural abundance ca. 9.5%) are not visible in frozen solution spectra.

The spin Hamiltonian parameters for (**1**) (see Table 1) are consistent with a six-coordinate Cr(I) environment and the strong ligand field splittings between the t_2g_ and e_g_ orbitals created by the carbonyl and phosphino ligands. This large splitting is ultimately responsible for the observed low-spin Cr(I) state (*S* = 1/2). In general, for any *d*^5^ system, the tetragonal distortion away from *O*_h_ to *D*_4h_ symmetry creates a ground state (*d*_xy_)^2^(*d*_xz_, *d*_yz_)^3^ or (*d*_xz_, *d*_yz_)^4^(*d*_xy_)^1^ electron configuration, depending on whether the ligand field splitting Δ_LF_ is positive or negative, respectively [16,26]. Simple ligand field arguments predict a (*d*_xz_, *d*_yz_)^4^(*d*_xy_)^1^ ground state for [Cr(CO)_4_(dppp)]^+^, as π-back donation to CO stabilises *d*_xz_, *d*_yz_ relative to *d*_xy_. As a result, the *d*_xz_ and *d*_yz_ orbitals lie just below the SOMO (i.e., E_xz−yz_ − E_xy_ is small and positive), while *d*_x2−y2_ will be empty and much higher in energy (i.e., E_x2−y2_ − E_xy_ is large and negative). One, therefore, predicts that *g*_xx_ and *g*_yy_ should be significantly larger than *g_e_* (producing a positive *g* shift), due to the admixture of the excited state resulting from the promotion of an electron from the doubly occupied *d*_xz,yz_ to the singly occupied *d*_xy_ orbital. For *g*_zz_, a negative *g* shift will be expected, arising from the promotion of the electron from *d*_xy_ into the empty *d*_x2−y2_ orbital. These estimated trends are all observed experimentally, with *g_e_* < *g*_Ʇ_ (*g*_xx_ ≡ *g*_yy_) = 2.068 and *g_e_* > *g*_||_ (*g*_zz_) = 1.991 (Table 1), agreeing with a *d*_xy_ ground state of [Cr(CO)_4_(dppp)]^+^ (**1**).

When this [Cr(CO)_4_(dppp)]^+^ complex (**1**) is exposed to UV radiation from a broad band light source (using the Labino (Vallentuna, Sweden) UVG 2.0 UV LED light with an output peak maximum at 365 nm) at room temperature, the deep blue colouration of the solution is immediately bleached, resulting in an almost colourless solution. The original EPR spectrum of the starting complex (Figure 1) is subsequently transformed into a new signal arising from a *trans*-[Cr(CO)_2_(dppp)_2_]^+^ complex [19]. This new *trans*-complex is the only observable EPR signal (with 100% contribution to the EPR spectrum) following room-temperature UV irradiation of (**1**) [19] (see Supplementary Materials, Figure S1). The photochemical transformation of (**1**) into the *trans*-complex, rather than the *cis*- complex, was in agreement with the known preference for the stabilization of *trans*- Cr(I) complexes compared to their *cis*- Cr(I) counterparts [27]. In contrast, when the UV irradiation was conducted at lower temperatures (typically 77–120 K), an intermediate species in this photochemical transformation was identified by CW EPR. This intermediate was assigned to a *mer*-[Cr(CO)_3_(*κ*^1^-dppp) (*κ*^2^-dppp)]^+^ complex (**2**), which subsequently reacts further under UV illumination and elevated temperatures into the *trans*-[Cr(CO)_2_(dppp)_2_]^+^ complex (**3**) (i.e., the step-wise transformation of (**1**) into (**2**) and finally to (**3**)).

This photoinduced reaction was also shown to be concentration-dependent (i.e., the mechanism operates under a bimolecular pathway [19]), and a distribution of all three (**1**–**3**) species was found by EPR depending on the concentrations of (**1**) used. In all cases, the observed contributions of species (**1**–**3**) in the low-temperature experiments were typically ca. 40% for complex (**1**) and ca. 60% for complex (**2**), whereas at room temperature, only 100% of complex (**3**) was observed. The spin Hamiltonian parameters of all three complexes are given in Table 1.

### 2.2. Evidence of the UV Wavelength Dependency in the Cr(I) Photochemistry

As stated above, using a broad-band UV-A light source, complex (**3**) was found to be the most dominant species identified in the EPR spectra at room temperature, with (**2**) only observed at low temperature. The latter complex (**2**) quickly transforms into (**3**) following prolonged UV exposure. We, therefore, considered whether any wavelength dependencies were exhibited and operative in this photochemical reaction. As a result, we used different UV sources, including a low-power LED light source (output 350–390 nm, peak emission at 365 nm) and a narrow-band tuneable light source (TLS), to probe this reaction.

**Scheme 1:**
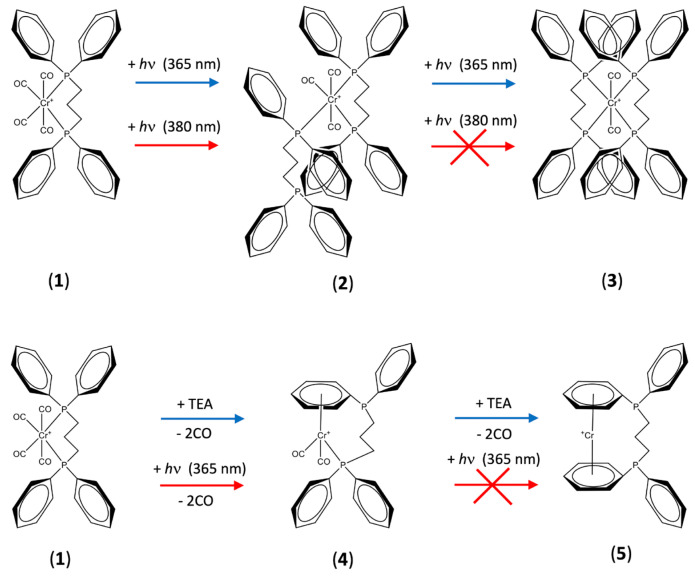
Upper—Photochemical transformations of [Cr(CO)_4_(dppp)]^+^ (**1**), into different complexes as a function of UV wavelength, including *mer*-[Cr(CO)_3_(*κ*^1^-dppp) (*κ*^2^-dppp)]^+^ (**2**) and *trans*-[Cr(CO)_2_(dppp)_2_]^+^ (**3**). Lower—Proposed transformations of [Cr(CO)_4_(dppp)]^+^ (**1**) into [Cr(CO)_2_(dppp-*η*^6^-arene)]^+^ (**4**) following exposure to TEA [17,18] or UV light.

**Figure 2:**
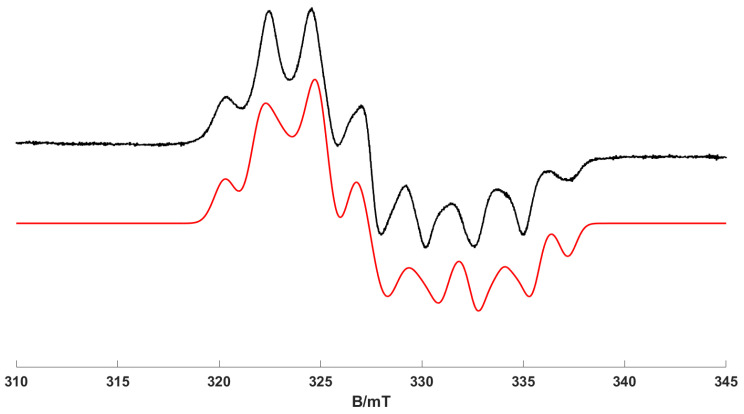
CW X-band EPR spectrum (recorded at 170 K) following room temperature UV irradiation (at 380 nm) of [Cr(CO)_4_(dppp)]^+^ (**1**) (using 1 mM of (**1**) dissolved in DCM). Experimental shown in black. The simulated spectrum (shown in red) was obtained using a single component of *mer*-[Cr(CO)_3_(*κ*^1^-dppp) (*κ*^2^-dppp)]^+^ (**2**).

When irradiating a 1 mM solution of [Cr(CO)_4_(dppp)]^+^ (**1**) in dichloromethane at 295 K using the LED source (output peak emission at 365 nm), the *trans*-[Cr(CO)_2_(dppp)_2_]^+^ complex (**3**) was exclusively observed in the room-temperature EPR spectrum (see Supplementary Materials, Figure S1). This result, obtained at 365 nm output peak emission, is consistent with our previously reported work [19]. However, when irradiating (**1**) at 295 K using the tuneable light source (TLS) at longer wavelengths (380 ± 2 nm), the resulting low-temperature (170 K) EPR spectrum is shown in Figure 2. Remarkably, and never observed before, the *mer*-[Cr(CO)_3_(*κ*^1^-dppp) (*κ*^2^-dppp)]^+^ complex (**2**) dominates the EPR spectrum, even after UV irradiation (380 nm) at room temperature (Figure 2). It should be noted that this experimental did produce some variability in the relative amount of (2) formed, and in some cases, the photochemical yield was lower (see Supplementary Materials, Figure S2). By comparison, when using a broad-band UV source (at 365 nm), the *trans*-[Cr(CO)_2_(dppp)_2_]^+^ complex was exclusively formed at the same temperature (Scheme 1). It should also be noted that when irradiating (**1**) using the tuneable light source (TLS) at even longer wavelengths (400 ± 2 nm), no changes were detected in the EPR spectrum; i.e., only the EPR signal of the starting (**1**) complex was observed. Crucially, these results clearly evidence, for the first time, a wavelength dependency to the observed photochemical transformations of [Cr(CO)_4_(dppp)]^+^ (**1**) (as illustrated in Scheme 1).

### 2.3. Selective Formation of the mer-[Cr(CO)_3_(κ^1^-dppp) (κ^2^-dppp)]^+^ Complex *(**2**)*

It is well known that the Cr-CO and Cr-P bonds are photolabile in a number of Cr(0) complexes, such as [Cr^0^(CO)_4_(L)_2_] (where L represents a bis(diphenylphosphino) ligand) [21,22,23,24]. A competitive photo-dissociation reaction must also be operative in the current Cr(I) complex (**1**), [Cr(CO)_4_(L)]^+^ (where L is also a bis(diphenylphosphino) ligand), such that the intramolecular exchange of P and CO ligands occurs between neighbouring Cr(I) complexes. This results in the ‘scrambling’ of the ligands that ultimately leads to the formation of complexes (**2**) and (**3**) (as shown in Scheme 1 and described elsewhere [19]). In particular, a transient bridged Cr-dppp-Cr dimer likely forms [19], resulting in the eventual exchange of a single dppp ligand from one Cr(I) centre to a nearby Cr(I) centre, already bearing a κ^2^-coordinated dppp ligand. This can then lead to the formation of the EPR-visible *mer*-complex (**2**). However, it appears from the current findings that at longer UV wavelengths (380 nm versus 365 nm), the final step of the photochemical transformation of (**2**) into (**3**) does not occur (i.e., from *mer*-[Cr(CO)_3_(*κ*^1^-dppp) (*κ*^2^-dppp)]^+^ in the *trans*-[Cr(CO)_2_(dppp)_2_]^+^ complex, which requires dissociation of the Cr-CO bond). Furthermore, at the even longer wavelengths used in this study, 400 nm, no photochemical transformation was detected at all.

We previously showed that the photochemically generated *mer*-[Cr(CO)_3_(*κ*^1^-dppp) (*κ*^2^-dppp)]^+^ (**2**) complex was unstable; leaving a solution of (**2**) to stand in the dark at 298 K for several hours resulted only in the observation of a weak residual signal assigned to the starting [Cr(CO)_4_(dppp)]^+^ complex. In other words, the photochemically generated (**2**) complex does not thermally transform into a kinetically inert complex (**3**). Indeed, even prolonging the irradiation time at 380 nm indefinitely (for several hours) does not result in the formation of the *trans*-[Cr(CO)_2_(dppp)_2_]^+^ (**3**). This step, requiring the photo-initiated breakage of a final Cr-CO bond in (**2**) and subsequent ligand rearrangement, can only be accomplished with higher-energy UV radiation (365 nm). These results demonstrate, for the first time, the potential selective control that can be achieved with these photochemically formed species. Therefore, it is clear that following room-temperature irradiation of (**1**) at 365 nm, the dominant signal observed in the EPR spectrum is due to (**3**), whereas for similar experimental conditions conducted with 380 nm irradiation, the dominant signal observed in the EPR spectrum is due to (**2**), and, finally, at 400 nm, only the starting unreacted (**1**) complex is observed throughout.

### 2.4. Formation of [Cr(CO)_2_(dppp-η^6^-arene)]^+^ Complex *(**4**)*

**Figure 3:**
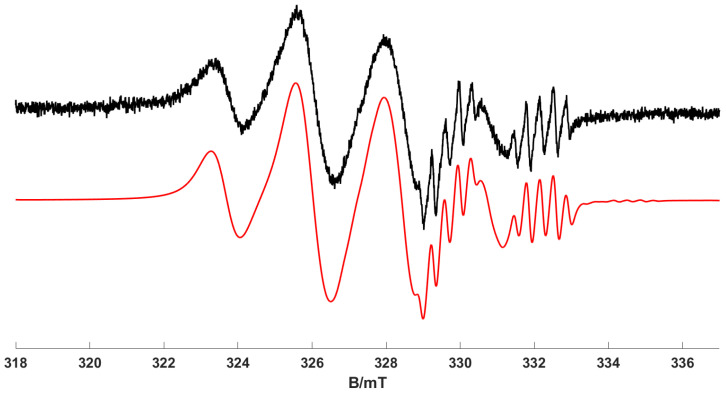
CW X-band EPR spectrum (recorded at 298 K) obtained following room temperature UV irradiation (at 380 nm) of [Cr(CO)_4_(dppp)]^+^ (**1**) (using 1 mM of (**1**) in DCM). Experimental spectrum shown in black. The simulated spectrum (shown in red) was obtained using weighted contributions of (**2**) *mer*-[Cr(CO)_3_(*κ*^1^-dppp) (*κ*^2^-dppp)]^+^ (95%) and (**4**) [Cr(CO)_2_(dppp-*η*^6^-arene)]^+^ (5%).

**Figure 4:**
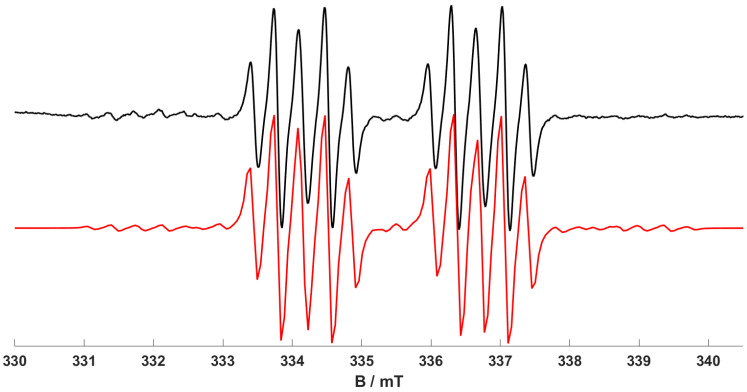
CW X-band EPR spectrum of the of [Cr(CO)_2_(dppp-*η*^6^-arene)]^+^ complex recorded at 295 K after thawing the frozen solution irradiated at 140 K (using 0.25 mM of (**1**) in DCM). Experimental shown in black, with corresponding simulation shown in red.

Figure 2 showed the low-temperature CW EPR spectrum of (**1**) following UV irradiation (380 nm) at room temperature. As discussed above, this EPR spectrum was dominated by the signal of complex (**2**), with only a small amount of complex (**1**) along with trace quantities of (**3**) observed. However, the resulting room-temperature isotropic EPR spectrum of complex (**1**) observed following UV irradiation at 380 ± 2 nm and 295 K is shown in Figure 3. The experimental spectrum is dominated by the isotropic features of complex (**2**) (noting that the EPR signal of complex (**1**) is not visible at 298 K due to fast relaxation [16]). A second species is also detected in the spectrum, as characterised by the additional series of resonances with narrower line widths at higher field (Figure 3). In addition, when lower concentrations of (**1**) (i.e., 0.25 mM) were irradiated at 140 K at 365 nm and the frozen solution quickly warmed to record the room-temperature EPR spectrum, the features from this second species become more apparent, as seen in Figure 4. The multipet hyperfine pattern in Figure 3 and Figure 4 can be assigned to the isotropic features of a ‘piano stool’ [Cr(CO)_2_(dppp-*η*^6^-arene)]^+^-type complex (**4**) (Table 2). An analogous species was already reported when a [Cr(CO)_4_(L)]^+^ complex (where L is a dppp or PNP ligand) was treated with small amounts of TEA, which leads to the direct formation of [Cr(L-bis-*η*^6^-arene)]^+^ (**5**) (see Scheme 1) [17,18].

Interestingly, this is the first time that the piano-stool complex (**4**) was observed following the photochemical transformation of (**1**). Previously, this complex was only observed by TEA activation [17,18] or by electrochemical treatment [25]. A plausible explanation for its photochemical formation at low concentrations of [Cr(CO)_4_(dppp)]^+^ is that the intermolecular exchange pathway associated with the ligand scrambling leading to (**2**) and (**3**) might be supressed when the concentration of [Cr(CO)_4_(dppp)]^+^ is low. This would favour the formation of paramagnetic species associated with unimolecular transformations rather than products of ligand scrambling. However, in the present work, there was no evidence to indicate the formation of the completely decarbonylated bis-arene sandwich complex previously reported with dppp and PNP ligand under TEA activation [17,18]. Furthermore, when a [Cr(CO)_4_(PNP)]^+^ was exposed to small quantities of TEA at low temperature, the piano-stool complex was observed to form first, and this slowly transformed into the bis-arene sandwich complex upon warming to room temperature. A similar transformation does not appear to occur in the presented photochemical reactions. Unlike the chemical reaction with TEA, which specifically removes CO ligands only and enable facile formation of [Cr(dppp-bis-*η*^6^-arene)]^+^ (**5**) (Scheme 1), the UV-induced photochemical reaction results in both Cr-CO and Cr-P photolysis so it is unlikely that the ‘piano-stool’ complex (**4**) can survive long enough to transform into the [Cr(dppp-bis-*η*^6^-arene)]^+^ (**5**) under UV irradiation conditions (Scheme 1).

**Figure 5:**
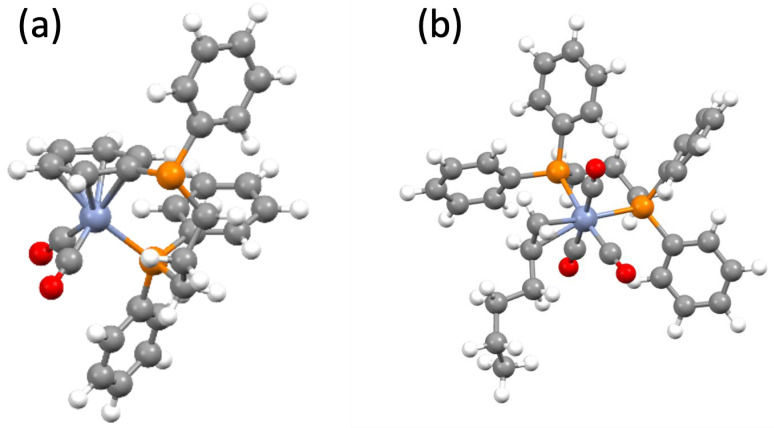
DFT geometry optimised structures of (**a**) [Cr(CO)_2_(dppp-*η*^6^-arene)]^+^ (**4**); and (**b**) [Cr(CO)_3_(dppp)(1-hexene)]^+^ (**6**).

In order to confirm the identity of the [Cr(CO)_2_(dppp-η^6^-arene)]^+^ complex (**4**), DFT calculations were performed on the energy-optimised structure of (**4**) (Figure 5). The resulting spin Hamiltonian values obtained are listed in Table 2 along with the corresponding simulated parameters. Whilst a reasonably good agreement exists between the experimental and calculated *g*_iso_ and ^31^P *a*_iso_ parameters, the agreement with the isotropic ^1^H couplings is less satisfactory (Table 2), and further improvements with other basis sets will be required.

Nevertheless, the current results demonstrate that, in addition to the dominant photochemical transformations of [Cr(CO)_2_(dppp)]^+^ (**1**) into *trans*-[Cr(CO)_2_(dppp)_2_]^+^ (**3**) through the intermediary *mer*-[Cr(CO)_3_(κ^1^-dppp)(κ^2^-dppp)]^+^ complex (**2**), other photochemical side reactions are also possible, including the formation of a new paramagnetic species identified as a ‘piano-stool’ [Cr(CO)_2_(dppp-*η*^6^-arene)]^+^ complex (**4**). Further experiments are underway to examine the feasibility of selectively controlling the nature and abundance of all these paramagnetic Cr(I) centres in the photochemical reactions, particularly where the suppression of catalytically inactive species (i.e., the bis-arene complexes) in ethylene oligomerisation is highly desirable.

### 2.5. Formation of [Cr(CO)_3_(dppp)(1-hexene)]^+^ Complex *(**6**)*

The above studies described so far have shown that the [Cr(CO)_4_(dppp)]^+^ complex displays complex photochemistry, dependent on the conditions of the experiment, including the sample concentration, temperature, and wavelength of UV light. The formation of complexes (**2**) and (**3**) is particularly sensitive to the concentrations, and these can only be formed photochemically (there is no route via treatment with TEA). On the other hand, it appears that the formation of the ‘piano-stool’ [Cr(CO)_2_(dppp-*η*^6^-arene)]^+^ complex (**4**), which was previously only formed via TEA activation [17,18], can also occur via photochemical treatment. This latter observation prompted us to investigate, using EPR, the feasibility of binding an olefin to the Cr(I) complex following UV irradiation, and, if successful, this could potentially provide a complementary route for the activation of (**1**) in ethylene oligomerisation catalysis using UV-A light, as opposed to TEA. Indeed, most recently, Chabbra et al. provided the first EPR evidence for the direct coordination of ethene to a Cr(I) complex, leading to a bis-ethene complex [Cr(C_2_H_4_)_2_(CO)_4_(PNP)]^+^, which is postulated to be a key intermediate in the metallacycle mechanism [28]. Hirscher et al. used pulsed EPR methods to characterise a Cr-hydrocarbyl species of considerable relevance to the ethylene tetramerization reaction [29]. Therefore, in the current study, 1-hexene was used as the model olefin substrate to explore this reaction with (**1**).

**Figure 6:**
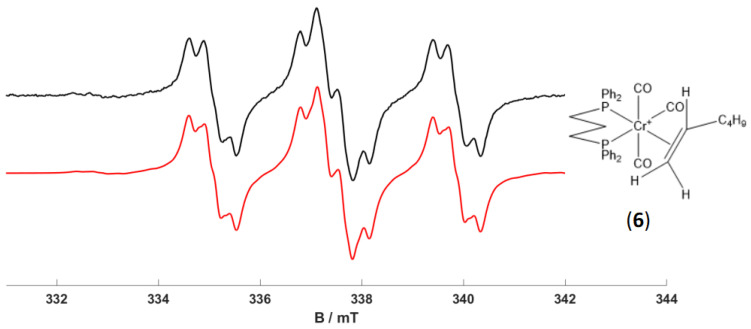
CW X-band EPR spectrum of (**1**) recorded at 298 K following brief (ca. 2 min) room temperature UV irradiation (365 nm) in the presence of excess 1-hexene (conditions: 0.33 mM of (**1**) dissolved in a 1:3 solution of DCM/1-hexene). Experimental shown in black, with corresponding simulation shown in red.

**Figure 7:**
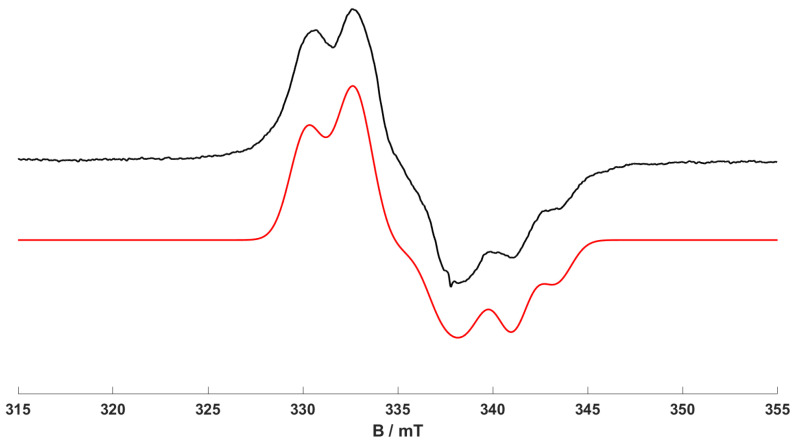
CW X-band EPR spectrum of (**1**) recorded at 140 K following brief (ca. 2 min) room temperature UV irradiation (365 nm) in the presence of excess 1-hexene (conditions: 0.33 mM of (**1**) dissolved in a 1:3 solution of DCM/1-hexene). Experimental shown in black, with corresponding simulation shown in red.

**Figure 8:**
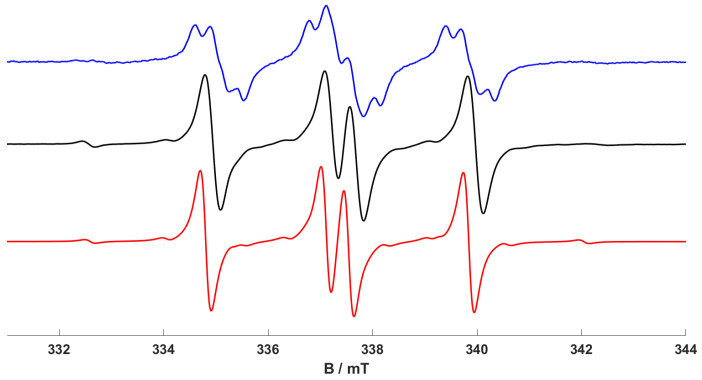
CW X-band EPR spectrum of (**1**) recorded at 298 K following brief (ca. 2 min) room temperature irradiation at 365 nm containing an excess of protic or fully deuterated 1-hexene (conditions: 0.33 mM of (1) dissolved in a 1:3 solution of DCM/1-hexene). Black trace: [Cr(CO)_3_(dppp)(1-hexene-d_12_)]^+^, Blue trace: [Cr(CO)_3_(dppp)(1-hexene-H_12_)]^+^ (previously shown in Figure 6). Experimental spectra shown in black and blue traces, with corresponding simulation shown in red.

A solution of [Cr(CO)_4_(dppp)]^+^ (**1**) in dichloromethane containing an excess of 1-hexene was prepared and exposed to UV-A irradiation (365 nm) at 295 K for 2 min. The irradiation time employed was kept short, specifically to prevent the over-formation of complex (**3**). Following these short UV irradiation times of (**1**) with excess 1-hexene, the resulting EPR spectra recorded at 298 K and 140 K are shown in Figure 6 and Figure 7. The simulations of both spectra revealed a quasi-axial *g* and *A*(^31^P) tensor, with hyperfine interactions coming from two inequivalent ^31^P nuclei from the dppp ligand. The inequivalency of the ^31^P hyperfine was also confirmed by DFT calculations. Smaller hyperfine splitting was also observed superimposed on the ^31^P pattern. This splitting was not previously observed for (**1**) so presumably arises from a ^1^H interaction with the 1-hexene. The nature of this ^1^H coupling was, therefore, corroborated by repeating the experiment using fully deuterated 1-hexene. The resulting spectrum (Figure 8) appears as a much simpler doublet of doublets, due to the two inequivalent ^31^P nuclei only, with no further splitting arising from the ^1^H super-hyperfine coupling. This result, therefore, fully confirms that the ^1^H nuclei observed are indeed the ^1^H on the sp^2^ C_1_ of the 1-hexene, having the double-bond directly coordinating to the Cr(I) centre, indicating the formation of a [Cr(CO)_3_(dppp)(1-hexene)]^+^ complex (**6**).

**Figure 9:**
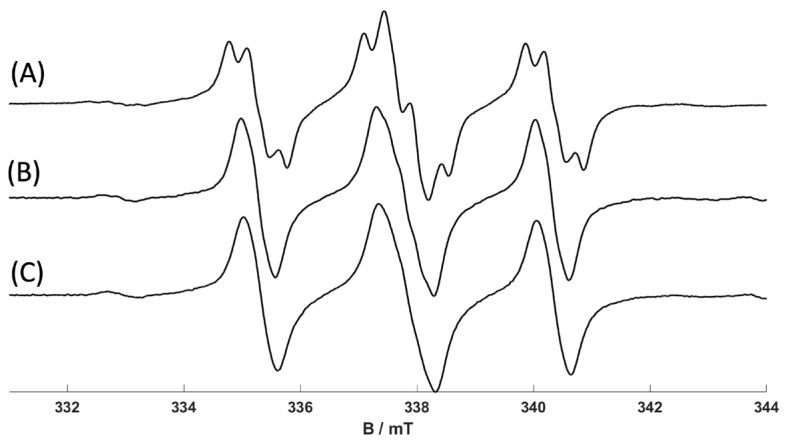
CW X-band EPR spectra of (**1**) recorded at 180–292 K following brief (ca. 2 min) room-temperature UV irradiation (365 nm) in the presence of excess hexene isomers, including (**A**) 1-hexene; (**B**) *cis*-2-hexene; and (**C**) *cis*-3-hexene (conditions: 0.33 mM of (**1**) dissolved in a 1:3 solution of DCM/hexene isomers). Some of these isotropic spectra were recorded at lower temperatures (e.g., 180 K) to extend the lifetime of the signal.

DFT was used to further examine the structure and stability of this 1-hexene coordinated complex, and the resulting calculated spin Hamiltonian parameters from the energy-optimised structure are listed in Table 3. The energy-optimised structure is shown in Figure 5b. Very good agreement was obtained between the experimental and calculated parameters, adding further confidence to the assignment and structure of complex (**6**) bearing the coordinated 1-hexene molecule. Owing to the bulky nature of the diphosphine ligand, coupled with the 1-hexene substrate, we were interested to examine the binding capabilities of other hexene isomers, notably *cis*-2-hexene, *cis*-3-hexene, *trans*-2-hexene, and *trans*-3-hexene. The resulting isotropic EPR spectra obtained using (**1**) with the hexene isomers are shown in Figure 9 and indicate that the less bulky *cis*- isomers can bind with the Cr(I) complex. The spectra obtained with *cis*-2-hexene and *cis*-3-hexene (Figure 9B,C) are analogous to that observed for [Cr(CO)_3_(dppp)(1-hexene)]^+^ (Figure 9A). The spectra were less well resolved and less stable, requiring slightly lower temperatures to obtain the isotropic EPR signal; nevertheless, the results clearly indicate the formation of [Cr(CO)_3_(dppp)(*cis*-2-hexene)]^+^ and [Cr(CO)_3_(dppp)(*cis*-3-hexene)]^+^. On the other hand, no equivalent EPR spectra were obtained using *trans*-2-hexene and *trans*-3-hexene, clearly indicating that they do not bind to the Cr(I) complex owing to the bulky substituents.

## 3. Experimental

### Synthesis

The experimental conditions used in the preparation of the air-sensitive [Cr(CO)_4_(dppp)]^+^ complex (**1**) were described in detail elsewhere [18]. In brief, the necessary manipulations in the preparation and handling of the complexes were carried out under a dry and inert atmosphere (N_2_ or Ar) using standard Schlenk-line and glovebox techniques. The dppp ligand and the silver tetrakis(perfluoro-tert-butoxy)aluminate (Ag[Al(OC(CF_3_)_3_)_4_]) were purchased from Sigma (Livonia, MI, USA) and Lolitec (Heilbronn, Germany), respectively. The neutral [Cr(CO)_4_(dppp)] and cationic [Cr(CO)_4_(dppp)]^+^[Al(OC(CF_3_)_3_)_4_]^−^ complexes were synthesised according to literature procedures [6]. The spectroscopic properties of these two compounds (^1^H, ^13^C, ^19^F and ^31^P NMR, IR, MS) were consistent with literature data [18]. 1-hexene and the other hexene isomers were purchased from Thermofisher (Norristown, PA, USA) and used as received. The fully deuterated 1-hexene-d12 was purchased from Cambridge Isotope Laboratories.

Sample preparation for EPR measurements: All sample preparations carried out in an Ar or N_2_ glovebox 4.63 mg of [Cr(CO)_4_(dppp)]^+^[Al(OC(CF_3_)_3_)_4_]^−^ were dissolved in 3 mL of dichloromethane (DCM) to obtain a solution of 1 mM of Cr complex (**1**) in DCM. The sample was stored at 253 K in a screwcap vial to prevent the solvent innate volatility; the samples are reasonably stable under these conditions over extended periods of time. For EPR analysis, Wilmad LPV-7 EPR tubes (Vineland, NJ, USA) bearing airtight caps were used and found to be reliable up to 24 h once outside the glovebox. The total volume used for EPR analysis was 150 µL; where dilution was necessary, an aliquot was taken from the stock solution inside the glovebox and the requisite amount of DCM added to give a total volume of 150 µL within the EPR tube. UV irradiation was performed using different light sources inside the EPR cavity. Great care was needed to correctly align the sample to the cavity slit and ensure that the entire sample volume was irradiated. UV irradiation was conducted using either a Labino UVG 2.0 Torch UV LED light source with an output power of 112 mW at the sample (50 nm bandwidth centred at 365 nm) or a Scitech Tuneable Light System (abbreviated TLS) for narrow-band irradiation at different wavelengths (0.2 nm bandwidth @300–700 nm).

EPR Instrumentation: The continuous-wave (CW) EPR spectra were recorded on an X-band Bruker (Billerica, MA, USA) EMX spectrometer operating at 100 kHz field modulation frequency, 1 Gauss field modulation amplitude, 1–10 mW microwave power, and equipped with a high-sensitivity cavity (ER 4119HS). EPR computer simulations were performed using the Easyspin (Version 5.2.35) [30] toolbox operating in the Mathworks Matlab environment.

Details of DFT calculations: The geometries of the investigated complexes were optimised using Turbomole [31] at the uBP86/def2-TZVP level of theory [32,33,34] and confirmed as a true minimum via harmonic frequency calculation. Hyperfine coupling and g tensors were calculated using ORCA [35] at the PBE0 level of theory [36], using the Barone’s EPR-II basis set for the light elements C and H [37], def2-TZVP(-f) basis set for P [34], and the Core Properties (CP) basis set (defined in ORCA for 1st row transition metals) for Cr [38].

## 4. Conclusions

We studied the photochemistry of the cationic [Cr(CO)_4_(Ph_2_P(C_3_H_6_)PPh_2_,)]^+^[Al(OC(CF_3_)_3_)_4_]^−^ complex (**1**) in solution using continuous-wave (CW) EPR spectroscopy. The photoinduced reactivity of this starting complex [Cr(CO)_4_(dppp)]^+^ transforms first into an intermediate *mer*-[Cr(CO)_3_(*κ*^1^-dppp)(*κ*^2^-dppp)]^+^ complex (**2**) and finally into the *trans*-[Cr(CO)_2_(dppp)_2_]^+^ complex (**3**) following exposure to UV-A light. The intermediate complex (**2**) is only visible following low-temperature UV radiation at 360 nm (typically 77–120 K), whereas room-temperature radiation reveals the presence of the more stable (**3**) only. However, complex (**2**) can be observed at room temperature, in the absence of the *trans*-complex (**3**), following UV irradiation at 380 nm, and this result highlights a wavelength dependency for this transformation for the first time. Furthermore, using lower concentrations of (**1**) (i.e., 0.25 mM versus 1 mM) in the UV photoirradiation studies, small quantities of the ‘piano stool’ [Cr(CO)_2_(dppp-*η*^6^-arene)]^+^-type complex (**4**) were also identified in the EPR experiments. There was no evidence for the formation of the [Cr(L-bis-*η*^6^-arene)]^+^ complex (**5**) in these UV irradiation experiments. UV irradiation of the [Cr(CO)_4_(Ph_2_P(C_3_H_6_)PPh_2_,)]^+^[Al(OC(CF_3_)_3_)_4_]^−^ complex (**1**) was also conducted in the presence of olefins, namely various isomers of 1-hexene. For the first time, a 1-hexene coordinated complex [Cr(CO)_3_(dppp)(1-hexene)]^+^ (**6**) was identified and characterised by EPR spectroscopy. These results illustrate the facile photochemistry of the Cr(I) complex and also highlight the need to better understand this photochemical reactivity owing to the importance of these catalysts for olefin oligomerisation and, hence, the potential use of UV radiation during catalyst activation.

## Figures and Tables

**Table 1:** Spin Hamiltonian parameters for the Cr(I) complexes (**1**–**3**) shown in Scheme 1.

Compound	*g*_1_	*g*_2_	*g*_3_	α /deg	β /deg	γ /deg	^31^P *A*_1_ /MHz	^31^P *A*_2_ /MHz	^31^P *A*_3_ /MHz	‘α /deg	‘β /deg	‘γ /deg
**1**	1.991	2.068	2.068	−180	90	60	−65 −65	−77 −77	−77 −77	−120 −60	160 160	120 60
**2**	1.984	2.030	2.049	15	60	180	−46 −70 −46	−50 −70 −50	−60 −83 −60	−120 0 90	85 90 60	−68 60 −180
**3**	1.970	2.024	2.024	90	117	180	−80 −80 −80 −80	−82 −82 −82 −82	−82 −82 −82 −82	−20 20 −20 20	120 120 120 120	45 120 45 120

Note: The Euler angles (α, β, γ) for rotation of the *g* frame with respect to the molecular frames, and the Euler angles (‘α, ‘β, ‘γ) for rotation of the *A* frame with respect to the *g* frame, are given above (in degrees).

**Table 2:** Experimental and DFT calculated isotropic spin Hamiltonian parameters (*g*_iso_ and *a*_iso_ for ^31^P and ^1^H) for the [Cr(CO)_2_(dppp-*η*^6^-arene)]^+^ complex (**4**).

[Cr(CO)_2_(dppp-*η*^6^-arene)]^+^	*g*_iso_	^53^Cr *a*_iso_ /MHz	^31^P *a*_iso_ /MHz	^1^H (*para*) /MHz	^1^H (*ortho*) /MHz	^1^H (*ortho*) /MHz	^1^H (*meta*) /MHz	^1^H (*meta*) /MHz
Experimental	2.0078	44	−72	20.2	9.5	9.5	<2	<2
DFT	2.02152	27	−107	0.8	3.2	2.5	−0.7	−2.6

**Table 3:** Spin Hamiltonian parameters (experimental and calculated via DFT) for the [Cr(CO)_3_(dppp)(1-hexene)]^+^ complex (**6**). The anisotropic *A* values for the two inequivalent ^31^P nuclei are given separately.

[Cr(CO)_3_(dppp)(1-hexene)]^+^	*g*_1_	*g*_2_	*g*_3_	^53^Cr *a*_iso_ /MHz	^31^P *A*_1_ /MHz	^31^P *A*_2_ /MHz	^31^P *A*_3_ /MHz	^1^H *a*_iso_ /MHz
Experimental	1.990	2.059	2.046	41	−74 −53	−81 −66	−77 −63	9 9 5
DFT	1.9789	2.0115	2.0176	36	−84 −68	−85 −70	−97 −80	9 11 8

## Data Availability

The data presented in this study are available in article and Supplementary Materials.

## Supplementary Materials

The following supporting information can be downloaded at: https://www.mdpi.com/article/10.3390/molecules29020392/s1.
