# Supplementary material for: A Continuous-Wave EPR Investigation into the Photochemical Transformations of the Chromium(I) Carbonyl Complex [Cr(CO)4bis(diphenylphosphino)]+ and Reactivity with 1-hexene
Source: Molecules. 2024 Jan 12;29(2):392. doi: 10.3390/molecules29020392 (PMC10819051; doi:10.3390/molecules29020392)
Supplement: Supplementary file 1 [file molecules-29-00392-s001.zip › molecules-2777578-supplementary.pdf]

A Continuous-Wave EPR Investigation into the Photochemical Transformations of the Chromium(I) Carbonyl Complex [Cr(CO)$_4$bis(diphenylphosphino)]$^+$ and Reactivity with 1-hexene

David Fioco [1,2,*], Andrea Folli [1], James Platts [1], Mario Chiesa [2] and Damien M. Murphy [1,*]

[1] School of Chemistry, Cardiff University, Main Building, Park Place, Cardiff CF10 3AT, UK; follia@cardiff.ac.uk (A.F.); platts@cardiff.ac.uk (J.P.)
[2] Dipartimento di Chimica e NIS Centre, Università degli Studi di Torino, via P. Giuria 7, 10125 Turin, Italy; mario.chiesa@unito.it
[*] Correspondence: fiocod@cardiff.ac.uk (D.F.); murphydm@cardiff.ac.uk (D.M.M.)

Supporting Information

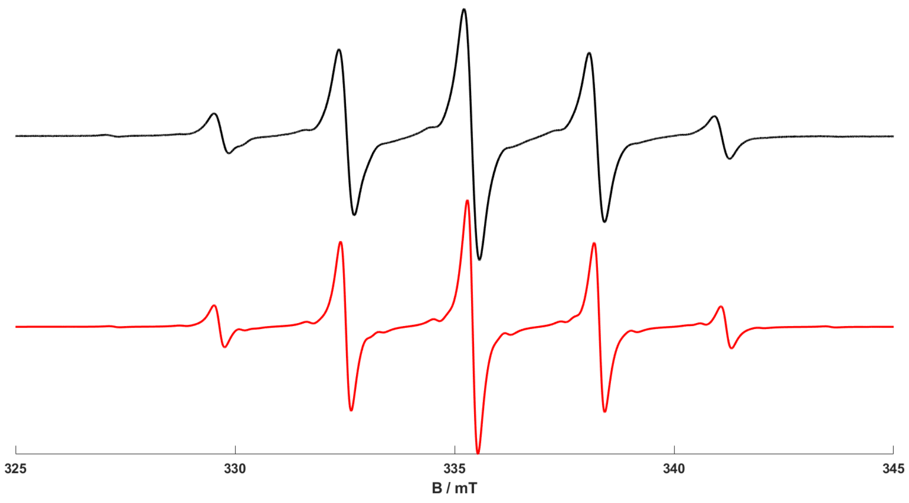

Figure S1. CW X-band EPR spectrum of the *trans*-$[Cr(CO)_2(dppp)_2]^+$ complex (**3**) recorded at 293 K (using 1 mM of (**1**) dissolved in dichloromethane) following 365 nm radiation at room temperature.

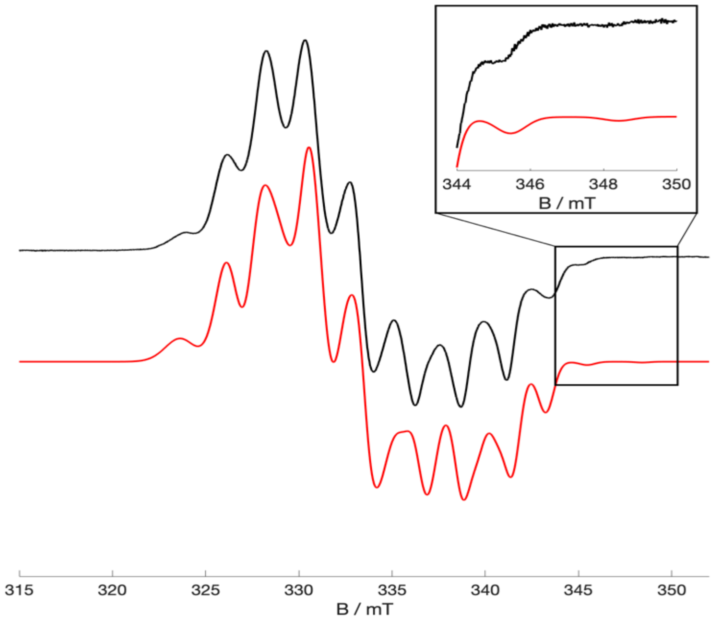

Figure S2. CW X band EPR spectrum (recorded at 140 K) obtained following room temperature UV irradiation (at 380 nm) of $[Cr(CO)_4(dppp)]^+$ (**1**) (using 1 mM of (1) dissolved in DCM). The simulated spectrum (shown in red) was obtained using weighted contributions of $[Cr(CO)_4(dppp)]^+$ (11%), *mer*-$[Cr(CO)_3(\kappa^1\text{-dppp})(\kappa^2\text{-dppp})]^+$ (86%) and *trans*-$[Cr(CO)_2(dppp)_2]^+$ (3%). This experiment is analogous to the result presented in Figure 2 (main

paper), except in that case the 'photochemical yield' of (2) was 100%, and in this case the yield was only 86%; this highlights the slight variability in the photochemical transformation at this wavelength.

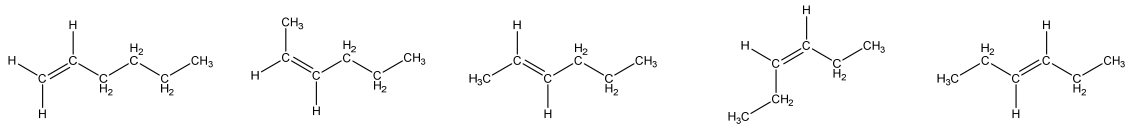

1-hexene *cis*- 2-hexene *trans*- 2-hexene *cis*- 3-hexene *trans*- 3-hexene

Figure S3. Schematic showing the different isomers of hexene studed here.
